# Identification of T cell dysfunction molecular subtypes and exploration of potential immunotherapy targets in BRAF V600E-mutant colorectal cancer

**DOI:** 10.1007/s12672-025-01930-8

**Published:** 2025-02-12

**Authors:** Tiefeng Gu, Haonan Qi, Jiaqi Wang, Liangwei Sun, Yongqi Su, Hanqing Hu

**Affiliations:** Department of Colorectal Surgery, The Second Affiliated Hospital of Harbin Medical University, 246 Xuefu Road, Harbin, China

**Keywords:** BRAF V600E, Colorectal cancer, Molecular subtypes, Immunotherapy, Machine learning, IDO1

## Abstract

**Background:**

Immunotherapy is an effective treatment for BRAF V600E-mutant colorectal cancer, but currently, only a few benefit from it. Therefore, exploring new immunotherapy strategies is essential.

**Methods:**

We obtained RNA sequencing data and clinical information of colorectal cancer patients from the TCGA and GEO databases. The impact of the BRAF V600E mutation on tumor microenvironment characteristics, gene expression, and signaling pathways was evaluated using bioinformatics approaches. Weighted gene co-expression network analysis (WGCNA) were used to identify core genes associated with T cell dysfunction. Consensus clustering was applied for subtype construction. Least Absolute Shrinkage and Selection Operator (LASSO) and Random Forest (RF) algorithms were employed to filter potential immunotherapy targets.

**Results:**

We found that BRAF V600E mutation has a complex impact on the immune profile of colorectal cancer. It increases immune cell infiltration and activates immune-related signaling pathways, yet it also severely restricts T cell function. We subsequently identified 39 core genes associated with T cell dysfunction and constructed subtypes of BRAF V600E colorectal cancer based on their expression profiles. Significant heterogeneity was observed between these subtypes in immune signaling pathway activity, immune infiltration patterns, immune phenotype scores, and mechanisms of resistance to immunotherapy. Ultimately, using machine learning algorithms and bioinformatics validation, we identified IDO1 as a potential immunotherapy targets for BRAF V600E-mutant colorectal cancer.

**Conclusion:**

This study constructed novel T cell dysfunction molecular subtypes for BRAF V600E-mutant colorectal cancer and identified IDO1 as a potential immunotherapy target, providing a new strategy for immunotherapy.

## Introduction

BRAF mutation is a key driver mutation in the development and progression of various cancers, including melanoma, colorectal cancer (CRC), non-small cell lung cancer (NSCLC), and others [1]. Approximately 5–15% of CRC patients harbor BRAF mutations, more than 90% of which is BRAF V600E mutation, leading to prolonged activation of the MAPK pathway and promoting tumor progression [2–4]. BRAF V600E mutation is correlated with unfavorable pathological characteristics of CRC, including poor differentiation, mucinous adenocarcinoma, signet ring cell carcinoma, and right-sided colon malignancies [5]. Patients with BRAF V600E-mutant CRC are more likely to develop extensive peritoneal metastases than liver-limited metastases, are less likely to undergo resection surgery, and have a higher risk of postoperative recurrence [6]. Additionally, BRAF V600E-mutant CRC patients typically exhibit a poor response to standard therapy, resulting in a median overall survival (OS) that is approximately half that of BRAF wild-type patients [7–9]. Although combination of MAPK-pathway-related targeted therapy improves prognosis in these patients, most still experience disease progression due to drug resistance [10, 11].

Immune checkpoint inhibition therapy is considered a promising treatment for various cancers, including CRC, due to its superior therapeutic efficacy and durable response [12, 13]. However, since cancer is a highly heterogeneous disease, the efficacy of immunotherapy varies not only across different cancer types but also among molecular subtypes of the same cancer. Studies have shown that oncogenic driver mutations can influence the effectiveness of immunotherapy. For example, KRAS mutation promotes immunotherapy resistance by reshaping the tumor microenvironment (TME) in NSCLC and CRC [14, 15]. Hence, understanding how oncogenic driver mutations regulate the TME is crucial, as it may aid in identifying potential immunotherapy targets and biomarkers in populations with specific oncogenic mutations, ultimately improving therapeutic outcomes. In addition to KRAS mutation, BRAF V600E mutation has also been shown to impact the TME in CRC. Jie Zhi et al. demonstrated that BRAF mutation can induce a local immunosuppressive microenvironment in CRC through exosomal long noncoding RNAs [16]. Another research found it can lead to increased immune cell infiltration in CRC [17]. However, these studies have certain limitations, such as small patient cohorts and the inability to exclude interference from BRAF wild-type/RAS mutant patients. More importantly, existing studies have not examined the impact of BRAF V600E mutation on the functional status of key immune cells in CRC, such as CD8 + T cells, which play a critical role in immunotherapy [18].

In this study, we analyzed the impact of BRAF V600E mutation on TME characteristics, gene expression levels and signaling pathway activation in CRC. Subsequently, we focused on evaluating the functional status of CD8 + T cells in BRAF V600E-mutant CRC and identified core genes associated with T cell dysfunction. Based on these results, we developed novel T cell dysfunction molecular subtypes of BRAF V600E-mutant CRC and characterized the immune-related features of each subtype. Importantly, using machine learning algorithms, we identified IDO1 as a potential immunotherapy target in BRAF V600E-mutant colorectal cancer, offering a new strategy for treatment.

## Materials and methods

### Data acquirement and processing

We obtained RNA sequencing data (TPM) and clinical information from the TCGA and GEO databases. A total of 45 BRAF V600E-mutant CRC patients and 235 BRAF wild-type CRC patients were included in the TCGA database after excluding patients with RAS mutations. Normalized expression matrix data and clinical information for GSE35896, GSE39084, GSE39582, and GSE75316 were downloaded using the “GEOquery” R package [19]. The four GEO expression matrices were merged using R software, and batch effects in the combined expression matrix were corrected using the “SVA” R package. In total, 73 BRAF V600E-mutant CRC patients and 344 BRAF/RAS wild-type CRC patients were included from the GEO database.

### Assessment of immune infiltration patterns

The immune score, stromal score, and tumor purity of CRC patients were assessed using the “ESTIMATE” R package [20]. “CIBERSORT” and “TIMER” algorithms were applied to assess various immune cell infiltration in CRC patients, conducted through the “IOBR” R package. [21–23]

### Identification and functional enrichment analysis of differentially expressed genes

The “DESeq2” and “limma” R packages were used to identify differentially expressed genes (DEGs) in BRAF V600E-mutant CRC patients [24, 25]. RNA sequencing data were analyzed using the “DESeq2” R package, while microarray data were processed with the “limma” R package. The filtering criteria included |Log2 Fold Change|> 1 and adjusted P value < 0.05. The intersection of the analyzed results of TCGA and GEO data was identified as DEGs. The “clusterProfiler” R package was utilized for Gene Ontology (GO) and Kyoto Encyclopedia of Genes and Genomes (KEGG) enrichment analysis of the DEGs [26].

### Calculation of T cell dysfunction score

We used the “IOBR” R package to calculate the T cell dysfunction score, which is based on three publicly available T cell dysfunction signatures and the “PCA” algorithm [27–29]. The effect of BRAF V600E mutation on T cell dysfunction scores was analyzed separately in the TCGA and GEO datasets.

### Identification and genetic correlation analysis of core genes associated with T cell dysfunction

A weighted gene co-expression network, constructed using the “WGCNA” R package, was employed to identify genes associated with T cell dysfunction in BRAF V600E-mutant CRC [30]. Genes in the top 50% of expression variability were included in the analysis, with the soft-threshold power set to 5. Core genes were then identified by cross-analyzing key module genes and DEGs. Correlation heatmaps and protein–protein interaction (PPI) networks were used to illustrate the intrinsic relationships among these genes.

### Construction and analysis of molecular subtypes of T cell dysfunction

T cell dysfunction core gene expression profiles of BRAF V600E-mutant CRC were extracted from GEO data. Clustering was performed on these profiles using the “ConsensusClusterPlus” R package [31]. The “clusterProfiler” and “GSEABase” R packages were utilized to perform Gene Set Enrichment Analysis (GSEA) of immune-related signaling pathways between subtypes. Results from CIBERSORT analyses were used to assess immune infiltration patterns across the different subtypes. The “IOBR” R package and the online database TIDE (http://tide.dfci.harvard.edu/) were used to calculate immunophenotypic scores and predict potential immunotherapy efficacy.

### Machine learning algorithm identifies potential immunotherapy targets

The least absolute shrinkage and selection operator (LASSO) and random forest (RF) algorithms were used to identify potential immunotherapy targets for BRAF V600E-mutant colorectal cancer. The "glmnet" R package was used to construct the LASSO model, and tenfold cross-validation was performed to select the λ value that minimized the error. Subsequently, we extracted the regression coefficients based on the optimal λ value and identified the feature genes corresponding to non-zero coefficients. The "randomForest" R package was used to train the RF model. Initially, we constructed a preliminary model with 500 trees and evaluated its performance under different tree numbers. After identifying the number of trees that minimized the error, we retrained the model using this optimal parameter. Upon completion of model training, we assessed the importance of each gene and selected those with importance scores greater than 2 as feature genes. The LASSO algorithm effectively reduces redundant features and compresses unimportant coefficients by incorporating L1 regularization, making it suitable for feature selection in high-dimensional data. The RF algorithm enhances model robustness by constructing multiple decision trees and introducing randomness, effectively identifying important features in complex nonlinear relationships. Ultimately, the intersection of the results from the two algorithms were identified as potential immunotherapy targets. Bioinformatics analysis was conducted for subsequent validation.

### Statistical analysis and visualization

All statistical analyses and result visualizations were conducted using R software (version 4.4.0). Comparisons between groups were performed using the Wilcoxon rank-sum test, while correlations were analyzed with Spearman's coefficient. A P value of < 0.05 was considered statistically significant unless otherwise specified.

## Results

### TME landscape of BRAF V600E-mutant CRC

**Fig. 1:**
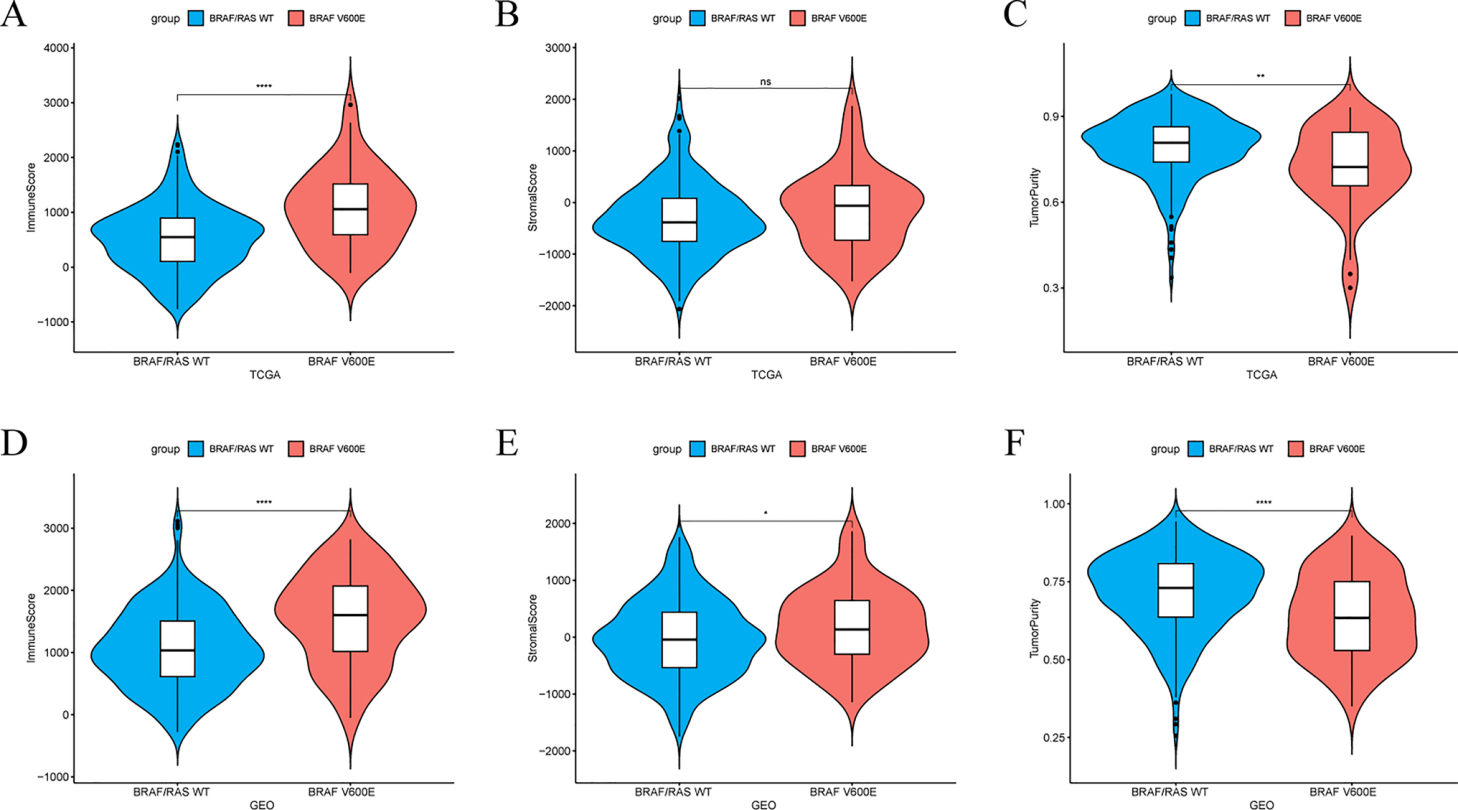
Impact of the BRAF V600E mutation on immune scores, stromal scores, and tumor purity in colorectal cancer. **A**–**C** TCGA dataset. **D**–**F** GEO dataset. *p < 0.05, **p < 0.01, ***p < 0.001, ****p < 0.0001, ns, not significant

**Fig. 2:**
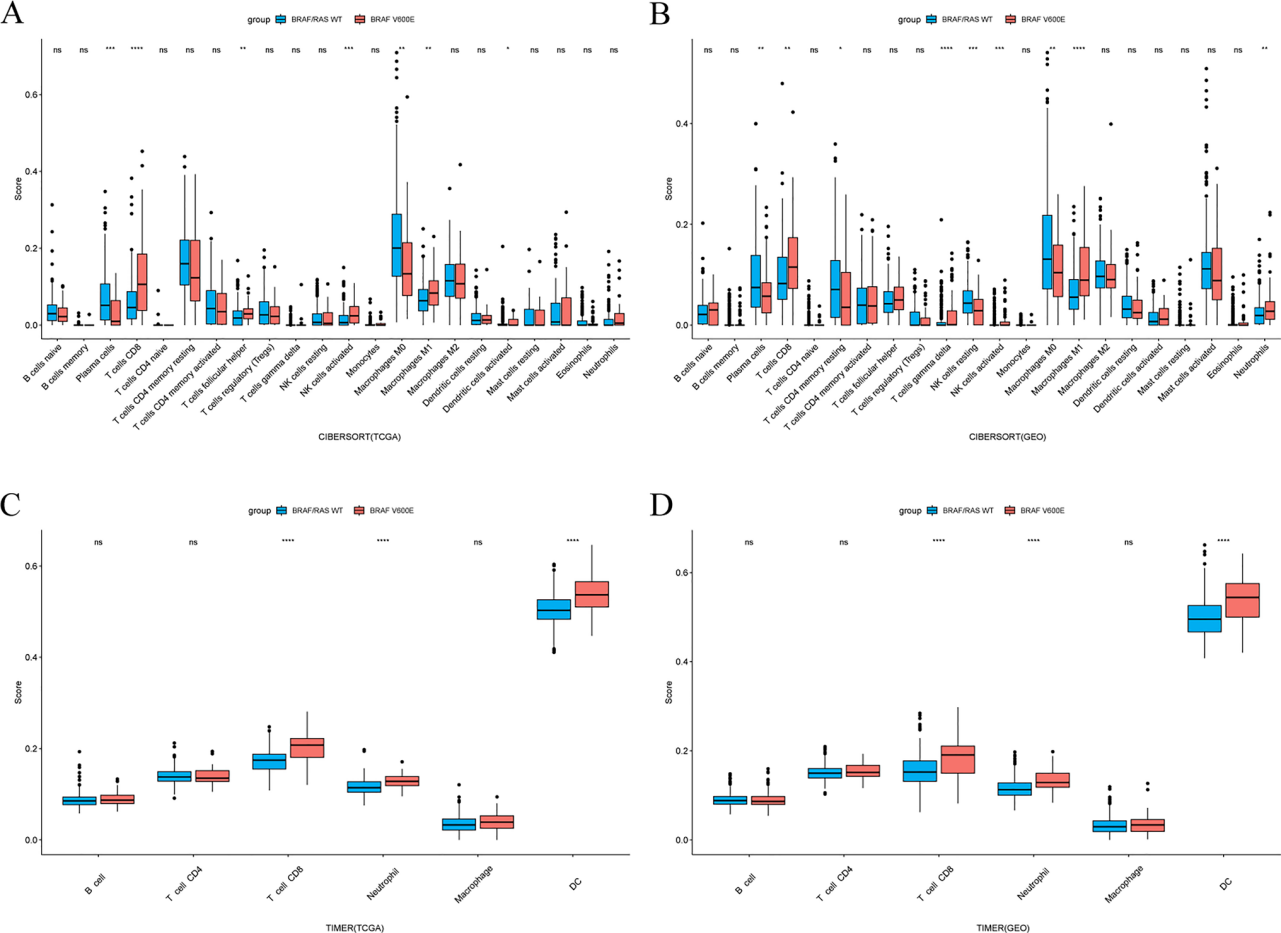
Impact of the BRAF V600E mutation on immune cell infiltration within the tumor microenvironment in colorectal cancer. **A**, **B** Results of immune cell infiltration analysis through the CIBERSORT algorithm. **C**, **D** Results of immune cell infiltration analysis through the TIMER algorithm. *p < 0.05, **p < 0.01, ***p < 0.001, ****p < 0.0001, ns, not significant

To elucidate the role of the BRAF V600E mutation in shaping TME of colorectal cancer, we independently analyzed TCGA and GEO datasets using multiple immune infiltration algorithms. Firstly, we observed that CRC patients with the BRAF V600E mutation exhibited significantly higher immune scores and lower tumor purity compared to wild-type CRC patients, while their stromal scores remained similar (Fig. 1A–F). Next, using the CIBERSORT and TIMER algorithms, we identified a significant increase in CD8 + T-cell infiltration in BRAF V600E-mutant CRC (Fig. 2A–D). Consistent with our findings, a recent study utilizing multiplex immunohistochemistry and multispectral imaging also reported elevated CD8 + T-cell infiltration in BRAF V600E-mutant CRC [32]. Furthermore, BRAF V600E-mutant CRC demonstrated significantly higher infiltration of M1 macrophages and lower infiltration of M0 macrophages. Collectively, these results suggest that BRAF V600E mutation is associated with enhanced immune infiltration in CRC.

### Identification and enrichment analysis of DEGs in BRAF V600E-mutant CRC

**Fig. 3:**
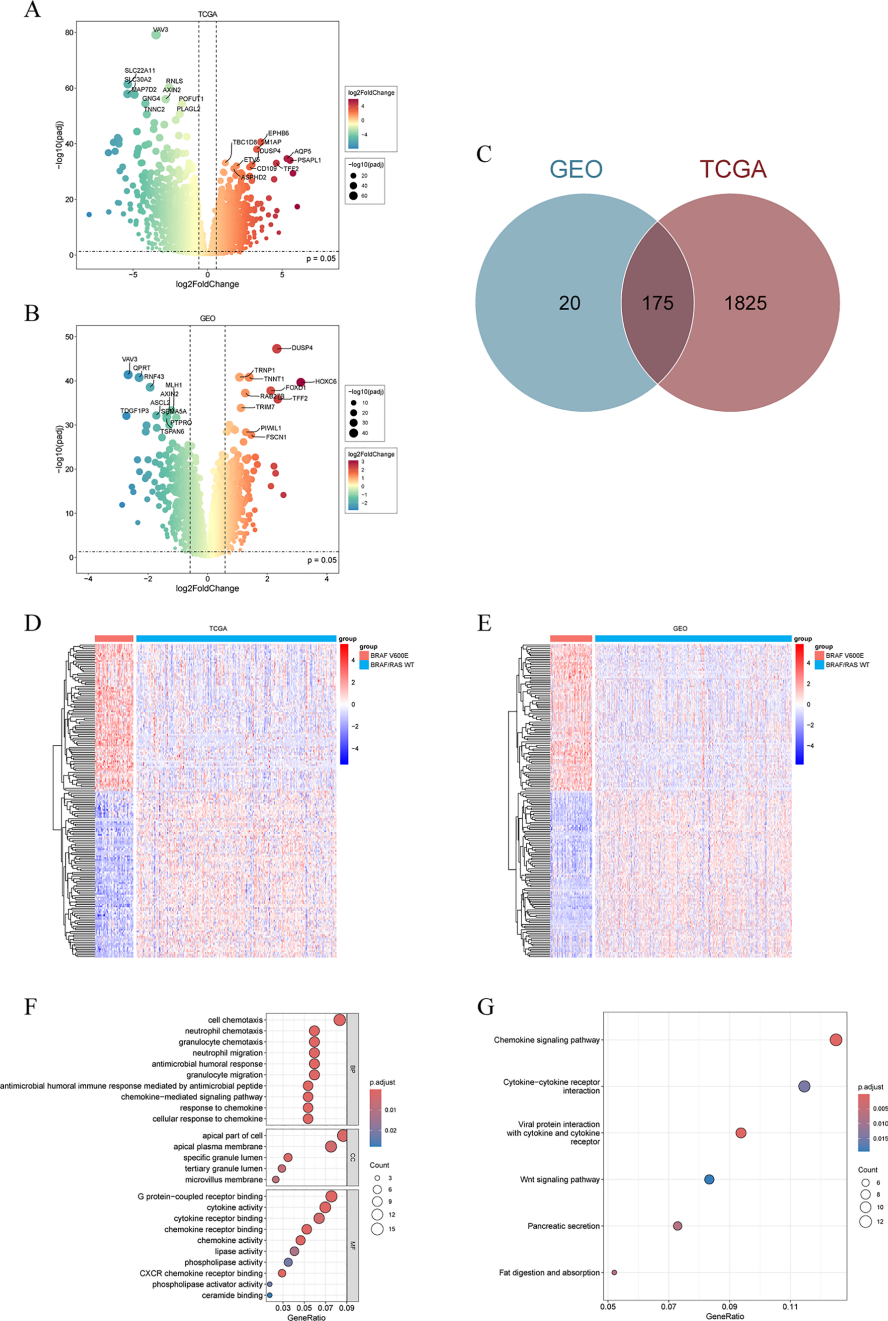
Identification and enrichment analysis of differentially expressed genes in BRAF V600E-mutant and BRAF/RAS wild-type colorectal cancer. **A** Volcano plot of differentially expressed genes in the TCGA dataset. **B** Volcano map of differentially expressed genes in the GEO dataset. **C** 175 differentially expressed genes were ultimately identified by intersection analysis. **D**, **E** Heatmap of differentially expressed genes for the TCGA and GEO datasets. **F**, **G** GO and KEGG enrichment analysis of differentially expressed genes

Differential expression analysis comparing BRAF V600E-mutant and wild-type CRC was independently conducted in the TCGA and GEO datasets. In the TCGA dataset, 1,900 genes were identified (Fig. 3A), whereas 195 genes were identified in the GEO dataset (Fig. 3B). By intersection analysis, 175 genes were identified as DEGs (Fig. 3C). Their expression patterns were visualized using heatmaps (Fig. 3D, E). GO and KEGG enrichment analyses demonstrated that these DEGs were primarily enriched in cytokine- and chemokine-related pathways, suggesting a potential role of the BRAF V600E mutation in modulating tumor immunity (Fig. 3F, G).

### Extent of T cell dysfunction and identification of core genes in BRAF V600E-mutant CRC

**Fig. 4:**
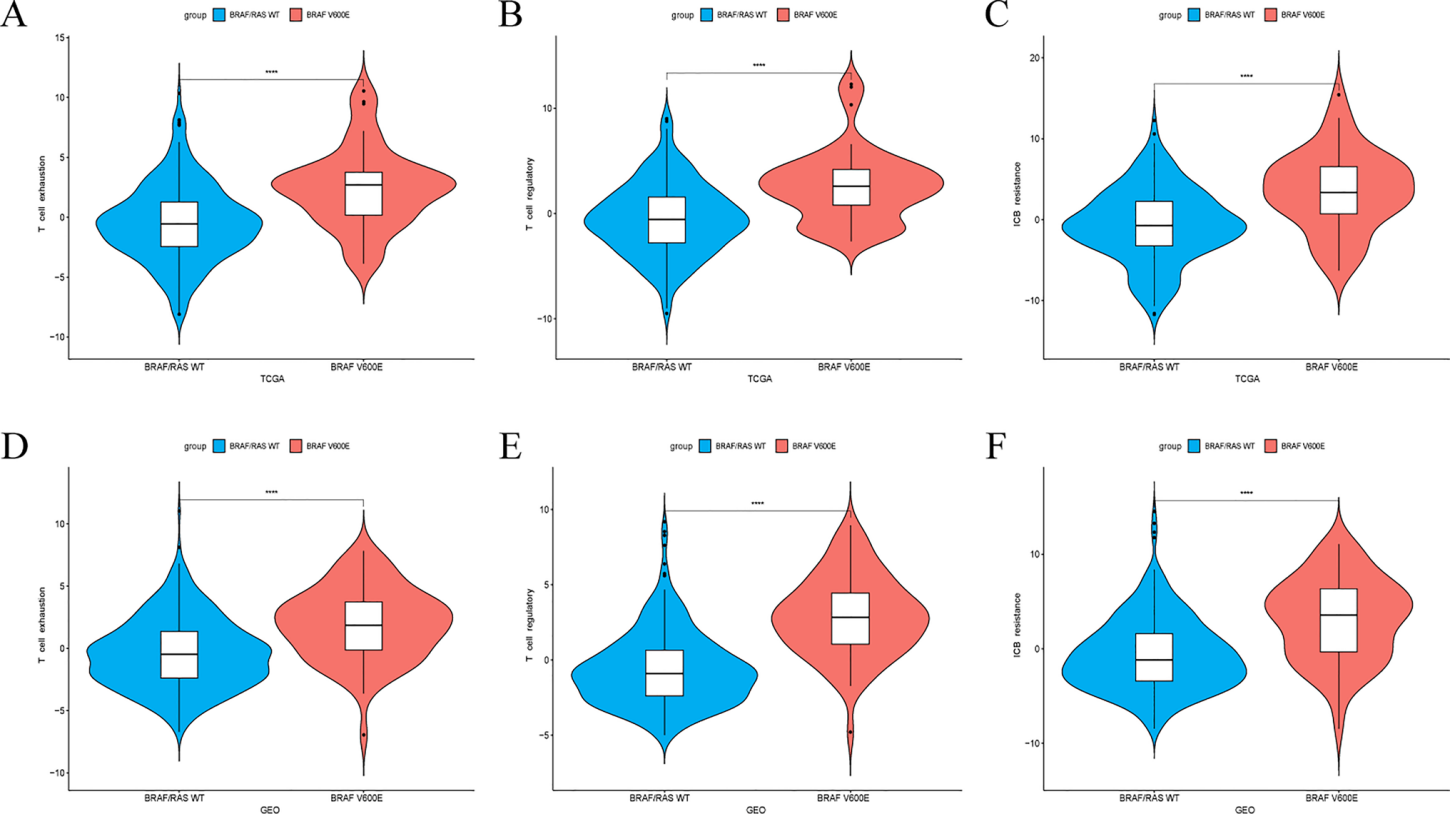
Impact of the BRAF V600E mutation on T cell functional status in the tumor microenvironment of colorectal cancer. **A**–**C** TCGA dataset. **D**–**F** GEO dataset. *p < 0.05, **p < 0.01, ***p < 0.001, ****p < 0.0001, ns, not significant

We evaluated the extent of T cell dysfunction in all CRC patients from the TCGA dataset and observed that BRAF V600E mutation was associated with significantly greater T-cell dysfunction (Fig. 4A–C). The same result was found in the GEO dataset (Fig. 4D–F). These findings suggest that BRAF V600E mutation may promote tumor progression by impairing the tumor-killing function of T cells.

**Fig. 5:**
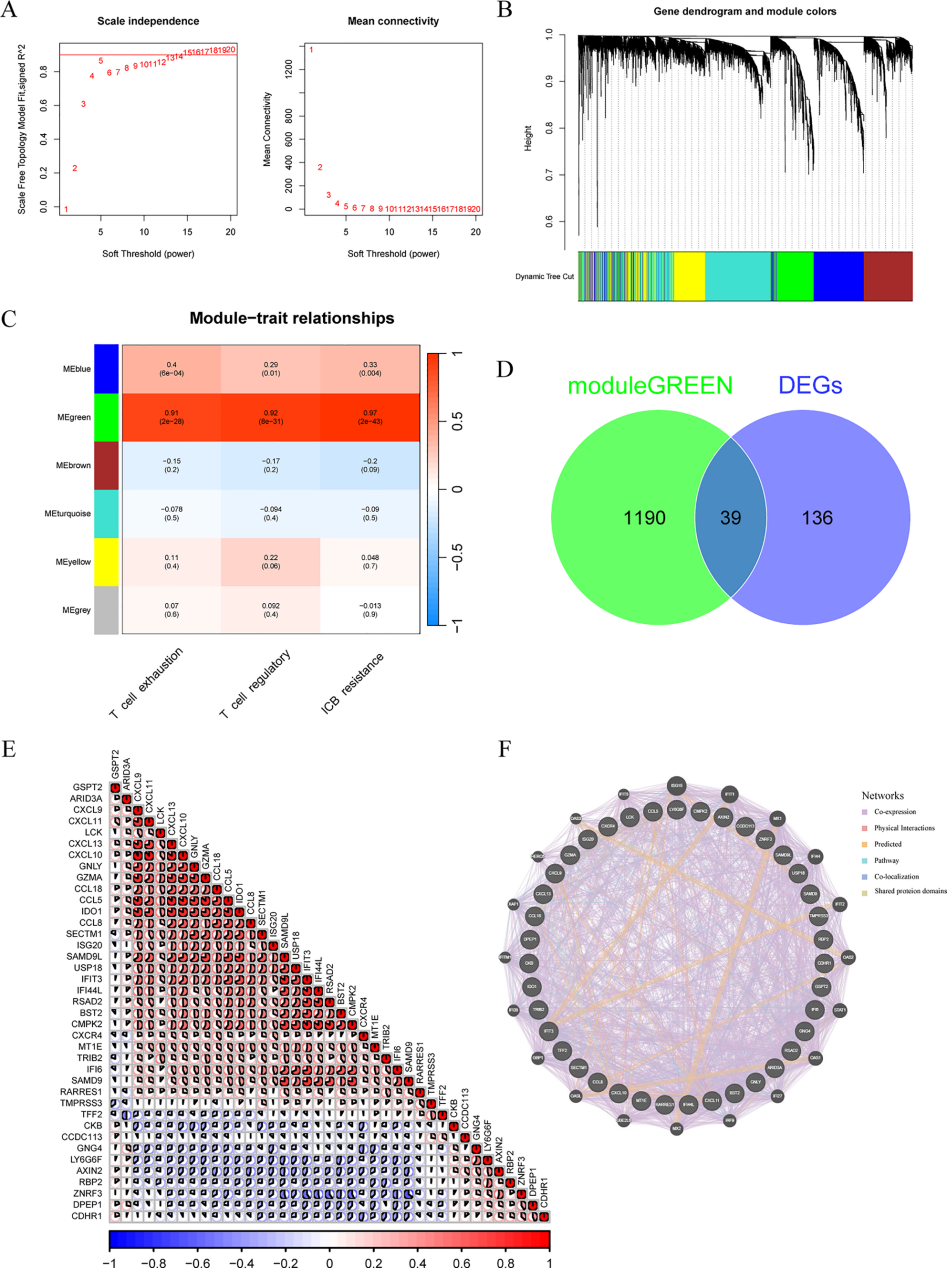
Identification and genetic correlation analysis of core genes associated with T cell dysfunction. **A** Determination of the soft thresholding power. **B** Dendrogram of differentially expressed genes clustered using the dissimilarity metric (1—TOM). **C** Correlation between gene modules and T-cell dysfunction scores. **D** Identification of core genes of T-cell dysfunction by cross-tabulation analysis. **E** Correlation analysis of core genes. **F** PPI network construction for core genes

Next, to identify core genes associated with T cell dysfunction in BRAF V600E-mutant CRC, we conducted WGCNA analyze using the GEO dataset. After filtering out low-expression genes, the top 50% of genes with the highest standard deviation were selected, resulting in 8,294 genes included in the analysis. A softpower of 5 was applied to construct the co-expression network, identifying six distinct modules (Fig. 5A, B). Correlation analysis between modules and T cell dysfunction related scores revealed that the green module exhibited the strongest correlation, comprising 1229 genes (Fig. 5C). Thus, the green module was selected as the key module for further analysis. By intersecting the green module genes with the DEGs, we identified 39 core genes associated with T cell dysfunction in BRAF V600E-mutant CRC (Fig. 5D). Correlation analysis revealed significant synergistic interactions among these core genes (Fig. 5E). PPI network analysis similarly demonstrated extensive co-expression and inter-gene interactions among these genes (Fig. 5F). These results suggest that these genes may constitute an interdependent regulatory network, functioning as a coordinated cluster, warranting further investigation into their roles and mechanisms.

### Construction of T cell dysfunction molecular subtypes in BRAF V600E-mutant CRC

**Fig. 6:**
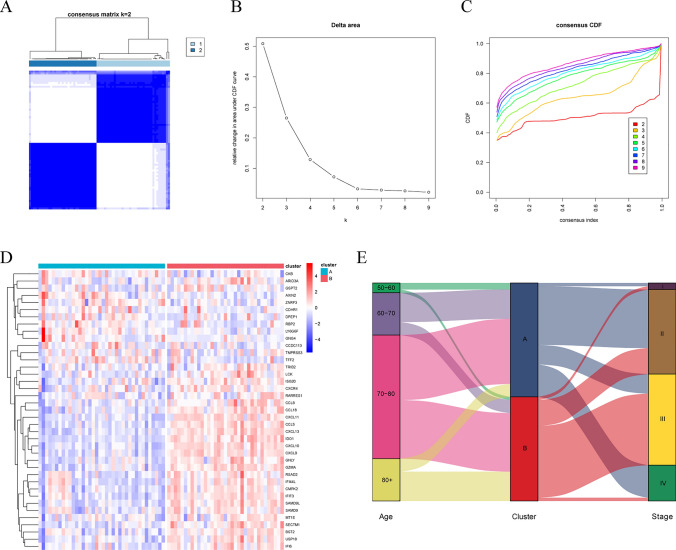
Identification of T cell dysfunction subtypes in BRAF V600E-mutant colorectal cancer and clinical characterization of the subtypes. **A** Consensus clustering matrix when k = 2. **B** Relative alterations in CDF delta area curves. **C** Consensus CDF curves when k = 2 to 9. **D** Core gene expression patterns across different subtypes of T cell dysfunction. **E** Clinical features of different subtypes

**Fig. 7:**
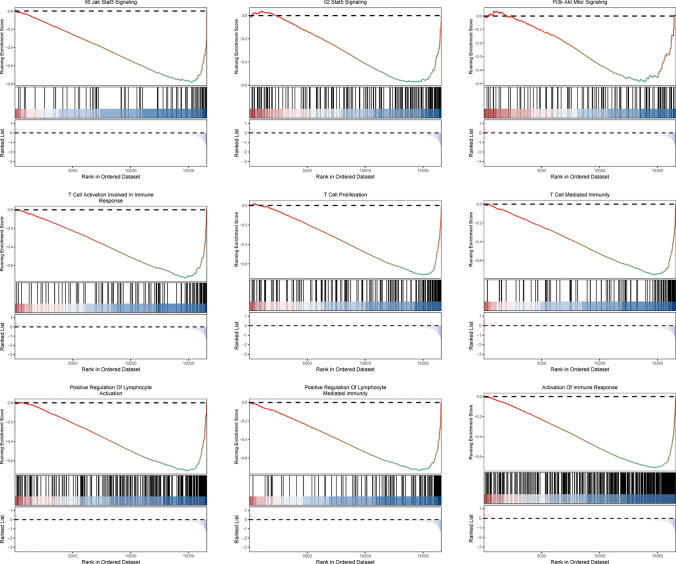
GSEA enrichment analysis of T cell dysfunctional subtypes

Using the “ConsensusClusterPlus” R package, we clustered BRAF V600E-mutant CRC patients in the GEO dataset based on core genes associated with T-cell dysfunction. The results indicate that the internal consistency and stability of the clusters are optimal when K = 2, prompting us to divide the patients into two clusters, Cluster A and Cluster B (Fig. 6A–C). Heatmap was used to demonstrate the differences in core gene expression patterns between clusters (Fig. 6D). We analyzed the correlation between clusters and age, as well as the TNM stage, and visualized the results using an alluvial diagram (Fig. 6E). The results showed that patients aged 50–70 years were more closely associated with Cluster A, those aged 80 years or older were predominantly linked to Cluster B, while patients aged 70–80 years exhibited roughly equal associations with both clusters. Meanwhile, we observed that patients in Cluster B were more frequently associated with advanced tumor stages. GSEA enrichment analysis revealed that immune-related signaling pathways and cell signaling regulatory pathways, including the IL2/STAT5, IL6/JAK/STAT3, PI3K-AKT, and lymphocyte-associated signaling pathways, were enriched in Cluster B (Fig. 7). These results suggest that Clusters A and B represent subtypes with distinct clinical and molecular features.

### Immune infiltration and immunotherapy efficacy prediction of molecular subtypes

**Fig. 8:**
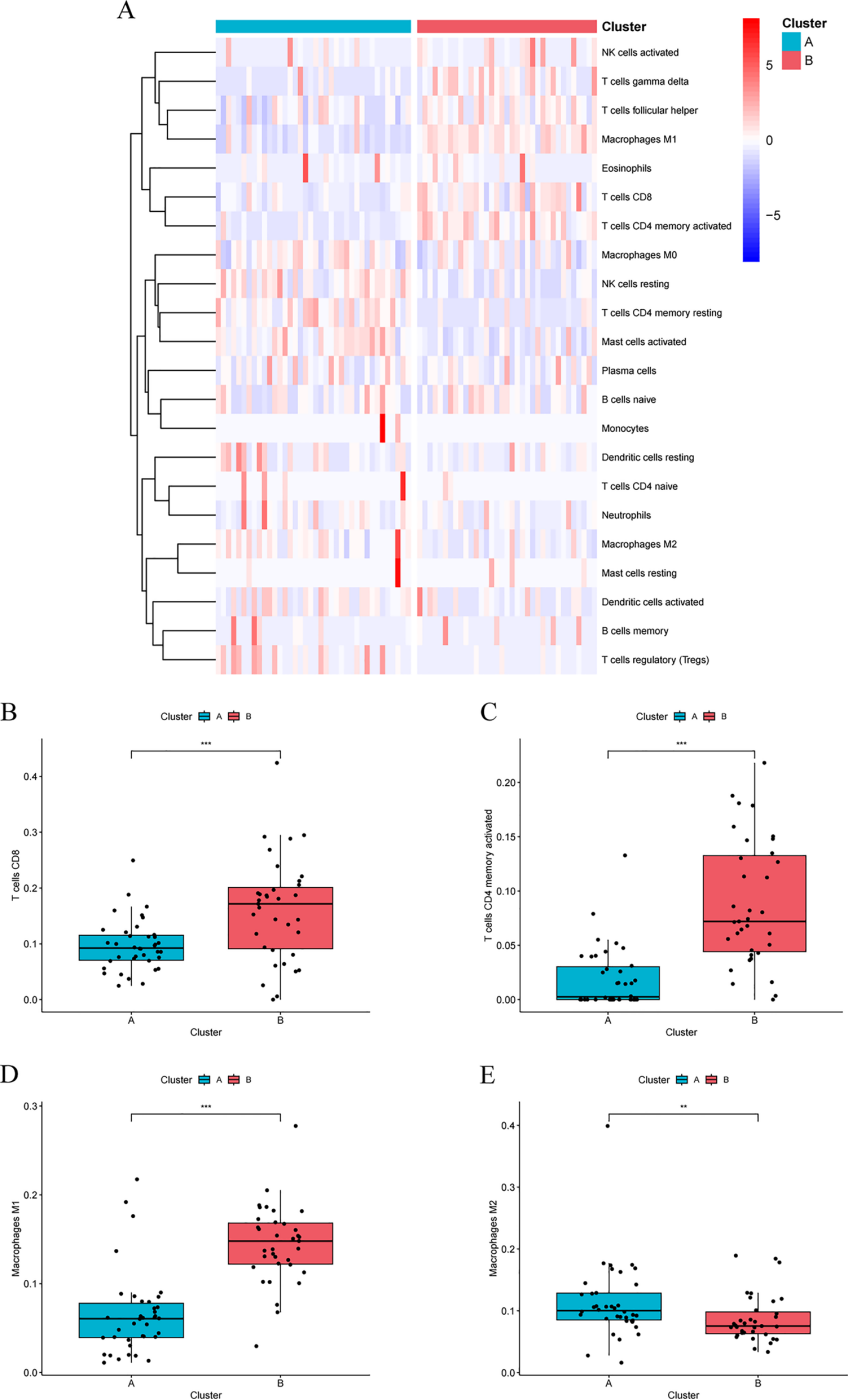
Differences in immune infiltration patterns between T cell dysfunction subtypes. **A** Overview of immune infiltration patterns across subtypes. **B** Infiltration differences of CD8 + T cells between subtypes.** C** Infiltration differences of activated memory CD4 + T cells between subtypes.** D** Infiltration differences of M1 macrophages between subtypes.** E** Infiltration differences of M2 macrophages between subtypes. *p < 0.05, **p < 0.01, ***p < 0.001, ****p < 0.0001, ns, not significant

**Fig. 9:**
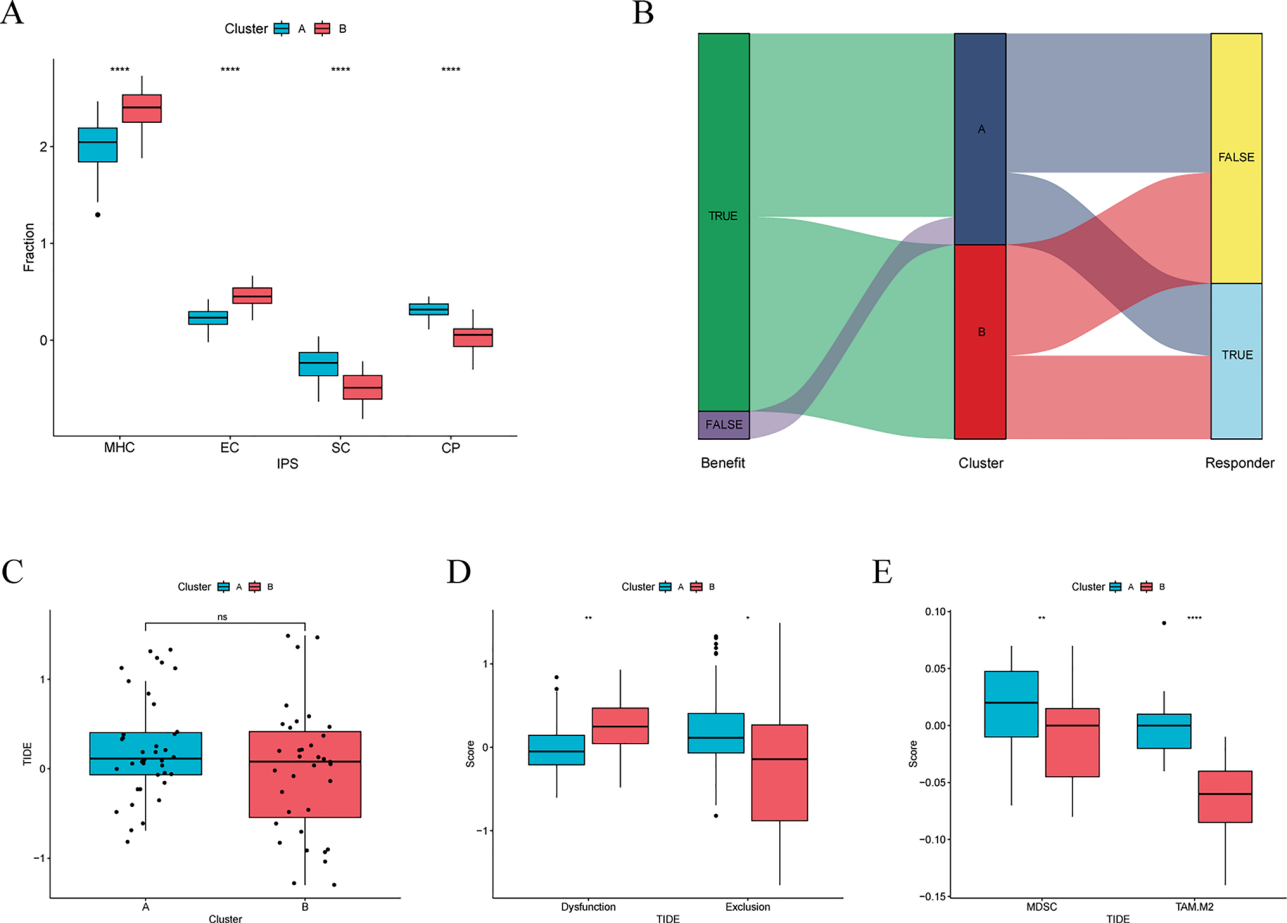
Differences in immune characteristics and predicted immunotherapy response among T cell dysfunction subtypes. **A** Major histocompatibility complex (MHC) molecules, effector cells (EC), suppressor cells (SC), and checkpoint (CP) scores of the different subtypes. **B** Proportion of individuals expected to benefit from or respond to immunotherapy. **C** TIDE scores for different subtypes. **D** Dysfunction scores and immune exclusion scores for different subtypes. **E** Immunosuppressive cell scores for different subtypes. *p < 0.05, **p < 0.01, ***p < 0.001, ****p < 0.0001, ns, not significant

To illustrate the immune characteristics between molecular subtypes, we first conducted immune infiltration analysis, the infiltration pattern was displayed by a heatmap (Fig. 8A). The results revealed significant heterogeneity in immune infiltration patterns between the two clusters. Compared to Cluster A, Cluster B exhibited significantly higher infiltration of CD8 + T cells, activated memory CD4 + T cells, and M1 macrophages, while the infiltration of M2 macrophages was significantly lower (Fig. 8B–E). Further comparison of immunophenotypic scores revealed that Cluster B had significantly higher scores for major histocompatibility complex (MHC) molecules and effector cells (EC) compared to Cluster A, whereas Cluster A showed significantly higher scores for suppressor cells (SC) and checkpoint (CP) compared to Cluster B (Fig. 9A). These findings suggest that Cluster B possesses greater immune potential and is more likely to benefit from immunotherapy.

Subsequently, we used the Tumor Immune Dysfunction and Exclusion (TIDE) to predict the effects of immunotherapy on different subtypes. However, the results were not entirely consistent with our expectations. Although Cluster B exhibited lower TIDE scores and a higher proportion of immunotherapy responders and beneficiaries compared to Cluster A, the difference between the two clusters was not statistically significant (Fig. 9B, C). This suggests that the expected efficacy of immune checkpoint inhibitor therapy may not differ significantly between the two clusters. To explore potential causes of this paradoxical phenomenon, we separately compared the dysfunction and exclusion scores across the different subtypes. The results showed that dysfunction scores were significantly higher in Cluster B compared to Cluster A (Fig. 9D). This explain why Cluster B failed to achieve the expected immunotherapy effect, despite having higher CD8 + T cell infiltration. With regard to exclusion scores, we observed that Cluster A was significantly higher than Cluster B, suggesting that the TME in Cluster A has a stronger immune exclusion function (Fig. 9D). A similar result was also obtained through the immunosuppressive cell scores, with myeloid-derived suppressor cell (MDSC) and M2 macrophage scores significantly higher in Cluster A (Fig. 9E). This is also consistent with previous results of immune cell infiltration analysis (Fig. 8A). Based on these results, we defined Cluster A as a subtype characterized by low CD8 + T cell infiltration and high immunosuppressive cell infiltration, making it less likely to benefit from immunotherapy. In contrast, Cluster B is defined as a subtype characterized by high CD8 + T cell infiltration and dysfunctional, with low immunosuppressive cell infiltration, presenting a significant potential to benefit from immunotherapy. Therefore, to translate this potential into immunotherapy benefits, we further investigated potential therapeutic targets to reverse the dysfunctional state of CD8 + T cells in Cluster B.

### Identification of immunotherapy targets in BRAF V600E-mutant CRC through machine learning algorithms

**Fig. 10:**
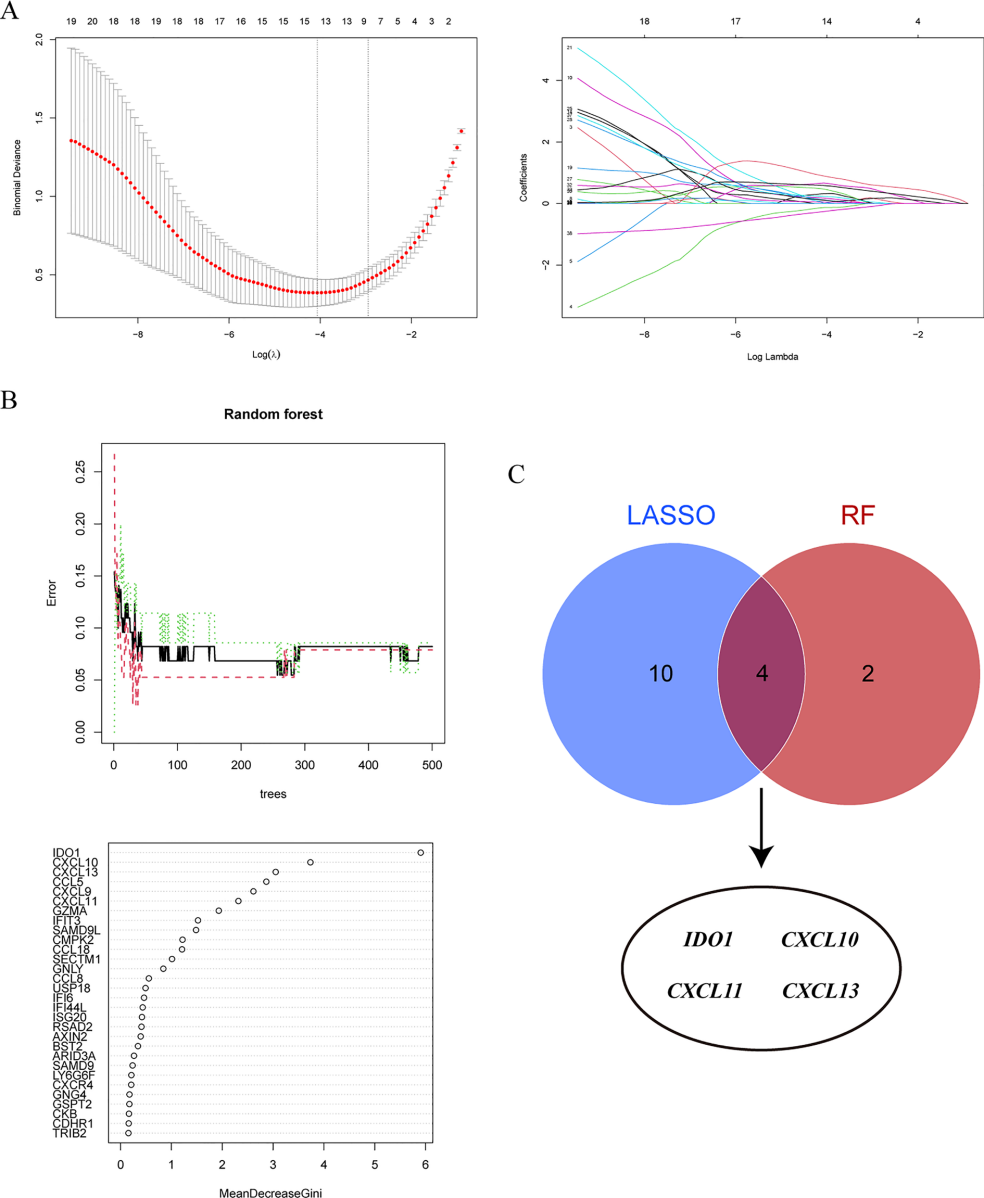
Identification of potential immunotherapy targets for BRAF V600E-mutant colorectal cancer using machine learning. **A** The Least Absolute Shrinkage and Selection Operator (LASSO) algorithm is used to filter feature genes.** B** Random forest (RF) algorithm is used to filter feature genes. **C** Identification of potential immunotherapy targets for BRAF V600E-mutant colorectal cancer through the intersection of results from two machine learning algorithms

To identify immunotherapy targets in BRAF V600E-mutant CRC, we analyzed T cell dysfunction core genes using multiple machine learning algorithms. The LASSO algorithm identified 14 genes closely associated with T cell dysfunction subtypes, including ARID3A, AXIN2, SECTM1, SAMD9, GSPT2, CMPK2, IFIT3, IFI6, CCL18, CXCL10, IDO1, CXCL11, TFF2, and CXCL13 (Fig. 10A). Meanwhile, the Random Forest algorithm highlighted six genes with feature importance scores > 2, including IDO1, CXCL10, CXCL13, CCL5, CXCL9, and CXCL11 (Fig. 10B). To refine the list of candidate target genes, we intersected the results of these algorithms and ultimately determined four genes C). Among them, IDO1 is a very attractive and research-worthy target for immunotherapy. As a key enzyme in human tryptophan metabolism, IDO1 catalyzes tryptophan degradation and promotes the accumulation of kynurenine [33]. By regulating this metabolic process, IDO1 can impair the function of CD4 + T cells, CD8 + T cells, and natural killer cells, and this inhibition only affects cells in an activated state [34]. Elevated IDO1 expression also promotes the activation and proliferation of regulatory T cells, dendritic cells, and myeloid-derived suppressor cells, thereby contributing to the formation of an immunosuppressive TME [35, 36]. Additionally, kynurenine accumulation activates the aryl hydrocarbon receptor (AHR), which subsequently disrupts immune function in the TME through multiple pathways [37, 38]. In conclusion, IDO1 plays a crucial role in immunosuppression and tumor immune evasion through the regulation of tryptophan metabolism, making it a valuable target for immunotherapy research. However, studies on the role of IDO1 in BRAF V600E-mutant CRC remain limited. In contrast, CXCL10, CXCL11, and CXCL14 primarily function in immune cell recruitment and immunosurveillance. Although essential for immune responses, their mechanisms of action as immunotherapy targets are less direct and effective compared to IDO1.Thus, we further explored the role of IDO1 in the immunotherapy of BRAF V600E-mutant CRC and evaluated its feasibility as a promising immunotherapy target.

### Exploring the role of IDO1 in BRAF V600E-mutant CRC

**Fig. 11:**
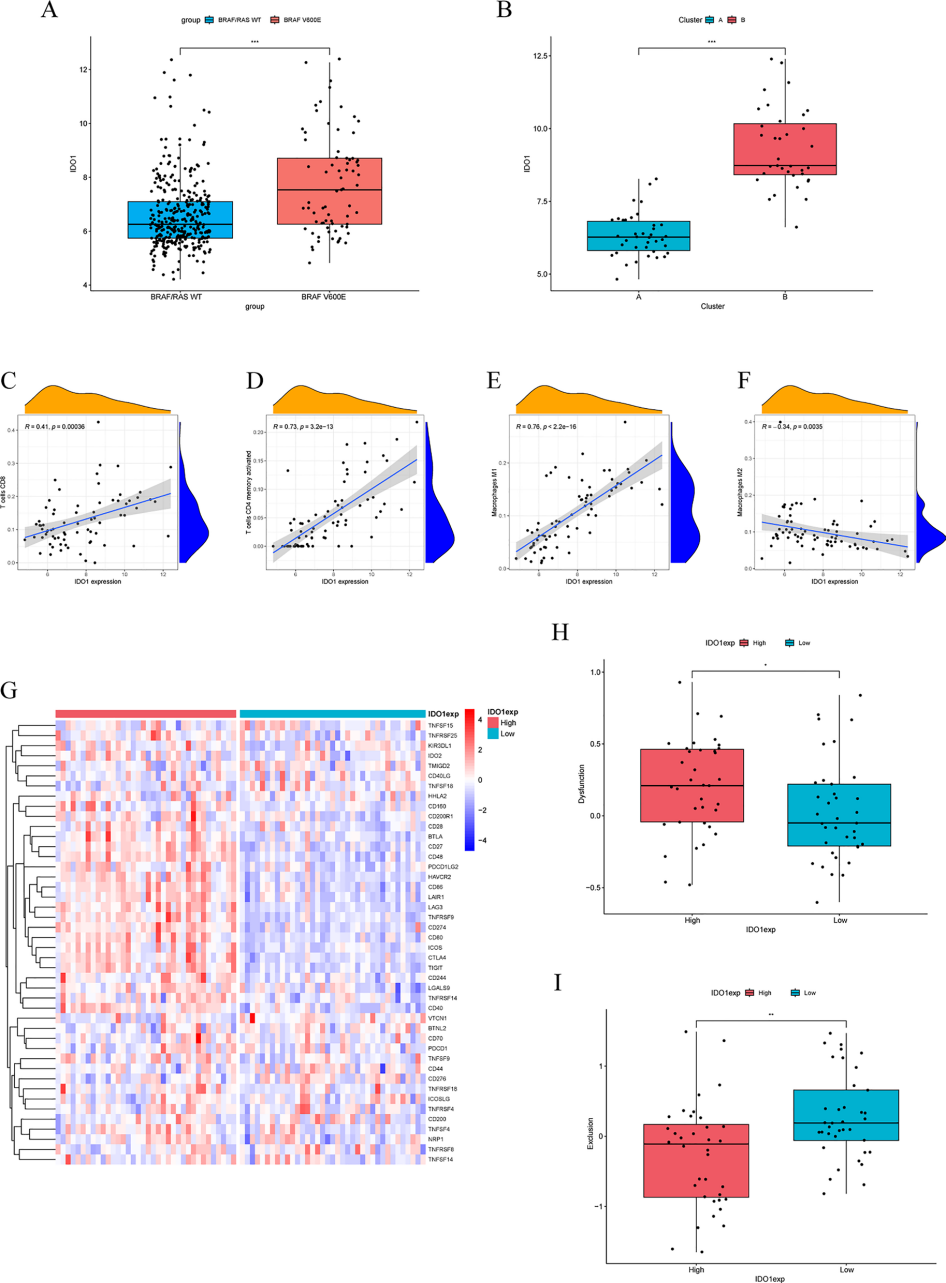
Expression and role of IDO1 in BRAF V600E-mutant colorectal cancer. **A** Impact of the BRAF V600E mutation on IDO1 expression levels in colorectal cancer. **B** Variations in IDO1 expression levels among T cell dysfunction subtypes. **C**–**F** Correlation between immune cell infiltration levels and IDO1 expression. **G** Differences in the expression patterns of immune checkpoints between IDO1 high and low expression groups. **H**, **I** Impact of IDO1 expression on dysfunction scores and immune exclusion scores. *p < 0.05, **p < 0.01, ***p < 0.001, ****p < 0.0001, ns, not significant

By analyzing gene expression level, we found that IDO1 expression was significantly higher in BRAF V600E-mutant CRC patients compared to wild-type CRC patients (Fig. 11A). In addition, the expression of IDO1 was significantly higher in Cluster B compared to Cluster A (Fig. 11B). These results suggest that in colorectal cancer, BRAF V600E mutation is associated with elevated IDO1 expression, which may play a critical role in driving increased T cell dysfunction. Correlation analysis revealed that IDO1 expression was significantly positively correlated with the infiltration levels of CD8 + T cells, activated CD4 + memory T cells, and M1 macrophages, while showing a significant negative correlation with M2 macrophage infiltration (Fig. 11C–F). We also observed that the BRAF V600E-mutant CRC patients with high IDO1 expression exhibited elevated levels of other immune checkpoints, such as LAG3 and CD274 (Fig. 11G). Dysfunction and exclusion score analyses further illustrated the function of IDO1 in BRAF V600E-mutant CRC, as its high expression was linked to significantly higher dysfunction scores and lower exclusion scores (Fig. 11H, I).

## Discussion

BRAF V600E-mutant colorectal cancer is characterized by high malignancy, resistance to standard treatments, and poor prognosis. Although MAPK pathway-targeted combination therapies have improved outcomes in this subgroup, most patients eventually develop drug resistance, resulting in disease progression [39]. Improving the prognosis for this population remains an urgent unmet clinical need, and immunotherapy is considered one of the most promising approaches due to its superior effectiveness and durable response. Therefore, investigating the impact of the BRAF V600E mutation on immunotherapy in colorectal cancer is crucial. A recent study demonstrated that the BRAF mutation significantly enhances cytotoxic T cell infiltration in the tumor microenvironment of colorectal cancer, indicating its potential as a favorable factor for immunotherapy [32]. However, clinical trial data reveal that BRAF mutations do not significantly influence the efficacy of immunotherapy in colorectal cancer patients [40]. This paradox highlights the need for a comprehensive investigation, which could provide novel insights into improving the efficacy of immunotherapy in BRAF V600E-mutant colorectal cancer. CD8 + T cells play a crucial role in anti-tumor immune responses; however, tumors can induce their dysfunction through various mechanisms, thereby impairing their immune function [41]. The primary goal of immunotherapy is to restore the anti-tumor immune function of CD8 + T cells by reversing T cell dysfunction. Consequently, T cell dysfunction plays a critical role in determining the efficacy of immunotherapy. However, its role in BRAF V600E-mutant CRC remains insufficiently studied. In this study, we analyzed the immune-related characteristics of BRAF V600E-mutant CRC and identified novel molecular subtypes based on genes closely associated with T cell dysfunction. Notably, we also identified IDO1 as a potentially immunotherapy target for patients with BRAF V600E-mutant colorectal cancer.

TME is a complex and highly heterogeneous ecosystem composed of various non-cancerous host cells, which plays a crucial role in cancer development and progression [42]. Additionally, the efficacy of immunotherapy is influenced by the composition and functional state of the TME [43–45]. In this study, we characterized the tumor microenvironment landscape of BRAF V600E-mutant colorectal cancer using RNA sequencing data, with the reliability of the results strengthened by independent analyses of different datasets. The analysis revealed significant heterogeneity in the tumor microenvironment composition between BRAF V600E-mutant and wild-type colorectal cancer patients. Overall, immune infiltration was significantly higher in BRAF V600E-mutant colorectal cancer, with notably elevated levels of CD8 + T cells, M0 macrophages, and M1 macrophages. This suggests that BRAF V600E mutation influences TME patterns in colorectal cancer, acting as a favorable factor for immune infiltration. Sofia Edin et al. evaluated immune cell infiltration using multiplex immunohistochemistry and multispectral imaging in a cohort of 257 colorectal cancer patients, finding results consistent with ours, thereby further confirming the reliability of our findings [32]. Additionally, we identified 175 genes with significantly altered expression in colorectal patients with BRAF V600E mutation. GO and KEGG enrichment analyses indicated that these genes are highly enriched in cytokine- and chemokine-related pathways, which play a crucial role in regulating immune response processes [46, 47]. The results of these analyses demonstrated that BRAF V600E mutation significantly alters the immune-related characteristics of colorectal cancer, providing a foundation for our further studies.

Besides the extent of T cell infiltration, the functional status of T cells is also closely linked to the efficacy of immunotherapy. In tumors with abundant T cell infiltration, T cell dysfunction plays a crucial role in facilitating tumor immune escape [48, 49]. Next, we analyzed the impact of BRAF V600E mutation on T cell functional status. In the GEO dataset, BRAF V600E-mutant CRC patients exhibited significantly higher T cell exhaustion scores, T cell regulation scores, and immune checkpoint blockade (ICB) resistance scores compared to wild-type CRC patients. Similarly, analysis of the TCGA dataset produced consistent results. These findings indicate that BRAF V600E mutation leads to a significantly greater degree of T cell dysfunction in CRC, severely compromising tumor immune function. Therefore, identifying the core genes in BRAF V600E-mutant CRC that regulate the functional state of T cells is crucial. Through the construction of a weighted gene co-expression network, we identified key modular genes associated with T cell dysfunction in BRAF V600E-mutant CRC. Subsequent intersection analysis with DEGs resulted in the identification of 39 core genes linked to T cell dysfunction. Gene correlation analysis and PPI network analysis suggested co-expression and synergistic effects among these genes, indicating that they collaboratively regulate the functional state of T cells in BRAF V600E-mutant CRC.

Colorectal cancer is a highly heterogeneous malignancy, exhibiting significant inter-individual differences in treatment responses and prognostic outcomes. As a result, stratifying the disease into subtypes with distinct molecular and clinical characteristics is crucial for precise treatment. Currently, constructing colorectal cancer molecular subtypes based on gene expression patterns has been demonstrated to be a reliable approach, with the consensus molecular subtype (CMS) classification being the most representative [50]. The recent study by Jun Xiang et al. established novel tumor microenvironment subtypes(TMEs), a classification framework that effectively identifies immunotherapy responders in colorectal cancer, further highlighting the value of this approach [51]. In BRAF V600E-mutant CRC, the study by David Barras et al. identified BM subtypes that exhibited significant differences in molecular characteristics and prognoses [52]. Our study constructed novel molecular subtypes of T cell dysfunction in BRAF V600E-mutant CRC, which demonstrated significant differences in immune-related and clinical features. Notably, substantial heterogeneity was observed in the immunotherapy resistance mechanisms among patients with different subtypes. GSEA enrichment analysis revealed that immune-related and cellular regulatory pathways were more active in Cluster B compared to Cluster A. Additionally, immune cell infiltration was more abundant in Cluster B. These findings suggest that Cluster B is an immunologically active subtype, further validated by the higher immunophenotypic scores observed in this subtype. Therefore, based on these results, we propose that Cluster B may exhibit better efficacy in response to immune checkpoint blockade therapy. However, the TIDE scores of the two patient clusters did not show statistically significant differences, suggesting that the efficacy of single-agent immune checkpoint inhibitor therapy may be similar between the subtypes. Given that the efficacy of tumor immunotherapy is influenced by multiple factors and mechanisms, it is essential to identify the key mechanisms underlying this paradoxical phenomenon. T cells, as the primary effectors of tumor elimination, play a crucial role in current tumor immunotherapy. Meanwhile, their abnormal functional state can significantly impact the efficacy of immunotherapy. T cell exhaustion was initially used to describe the marked reduction in T cell function observed in patients with chronic viral infections and is now also applied to characterize a state of severe T cell dysfunction in advanced progressive tumors [53]. T cell exhaustion impairs the effective restoration of anti-tumor immune function and is therefore commonly regarded as a key immunotherapy resistance mechanism [54]. Other factors within the TME, such as myeloid-derived suppressor cells, tumor-associated macrophages, and cancer-associated fibroblasts, can also limit the efficacy of tumor immunotherapy [42]. Notably, further analysis revealed significantly higher dysfunction scores and lower exclusion scores in Cluster B, indicating that T cell dysfunction is a key factor limiting the efficacy of immunotherapy. Some tumor patients exhibit resistance to immunotherapy because PD-1/PD-L1 monotherapy is ineffective in reversing T cell dysfunction [55]. We propose that this is the primary reason why patients in Cluster B fail to achieve the expected efficacy from immunotherapy. Screening for valuable immunotherapy targets and exploring immune-combination therapy options can effectively improve the efficacy of immunotherapy in this population. Based on T cell dysfunction molecular subtypes, we explored potential immunotherapy targets for BRAF V600E-mutant CRC using machine learning algorithms.

Machine learning is increasingly applied in cancer study, offering researchers valuable insights in cancer diagnosis, patient prognosis prediction, and enhanced treatment planning [56]. In this study, we identified IDO1 as a biomarker to distinguish T cell dysfunction subtypes in BRAF V600E-mutant CRC through LASSO and random forest algorithms. IDO1 is a key immunosuppressor in the tumor microenvironment. Previous studies have shown that IDO1 can reduce tryptophan levels and increase tryptophan metabolite levels, primarily kynurenine, in the tumor microenvironment by modulating the tryptophan metabolic pathway, resulting in dysfunction and diminished antitumor effects of CD8 + T cells [57, 58]. Despite the critical role of IDO1 in tryptophan metabolism and immunosuppression, clinical trials of its inhibitors have yielded disappointing results [59]. The ECHO-301/KEYNOTE-252 trial assessed the efficacy of epacadostat, an IDO1 inhibitor, in combination with pembrolizumab for various solid malignancies. Although this regimen showed higher objective response rates and potent anti-tumor effects in phase I/II, it failed to demonstrate further success in phase III [60, 61]. The failure of this pivotal trial highlights the potential complexity of IDO1-targeted therapy and has prompted researchers to deeply consider the underlying causes of failure. Selecting appropriate inhibitors is important for improving clinical outcomes, and clinical trials evaluating the efficacy of drugs such as Linrodostat (another IDO1 inhibitor), HTI-1090 (an IDO/TDO inhibitor), and others are currently underway [62, 63]. More effective inhibitors will also need to be developed in the future to address the limitations of current IDO1 inhibitors and enhance the clinical applicability of this therapeutic target. Meantime, it is crucial to dynamically monitor enzyme activity and tryptophan metabolite concentrations at the target site, as this data is essential for determining the appropriate drug dose and evaluating efficacy. In addition, the safety and toxicity of IDO1 inhibitors must be carefully evaluated. As IDO1 inhibitors target tryptophan metabolism, a normal physiological process in the human body, they may lead to serious side effects due to off-target effects [64]. Future development of IDO1 inhibitors should prioritize enhancing selectivity and specificity to improve safety. If these aspects can be improved, the strategy of utilizing IDO1 inhibitors for tumor treatment may yield satisfactory clinical outcomes. Combination therapy targeting IDO1 and immune checkpoints is also a focus of future research. Although some clinical trials have not yielded satisfactory results, breakthroughs in this therapeutic strategy are anticipated through the selection of appropriate patient populations, immune checkpoint inhibitors, dosages, and timing of immunotherapy.

Our study demonstrates for the first time that the BRAF V600E mutation significantly elevates IDO1 expression in colorectal cancer. Moreover, We further identified patients with high IDO1 expression by constructing T cell dysfunction subtypes. These results provide valuable guidance for identifying populations suitable for treatment with IDO1 inhibitors. Consistent with previous studies, we observed that IDO1 exerts a potent immunosuppressive effect in BRAF V600E-mutant CRC, inhibiting T cell antitumor function. These results suggest that IDO1 is a key contributor to T cell dysfunction in BRAF V600E-mutant CRC. Therefore, we demonstrate that targeting IDO1 to reverse T cell dysfunction could be an effective strategy for enhancing the efficacy of immunotherapy in BRAF V600E-mutant CRC. In addition, this study revealed that the elevated expression of IDO1 in BRAF V600E-mutant CRC is accompanied by increased expression of other immune checkpoints (e.g., LAG3), offering insights for developing novel combination therapy strategies.

However, this study has certain limitations. First, our data were derived from public databases and require further validation using real-world patient data. Additionally, further molecular biological experiments are needed to confirm the expression and mechanism of IDO1 in BRAF V600E-mutant CRC. Furthermore, relevant clinical trials are required to evaluate the efficacy of IDO1 inhibitors in BRAF V600E-mutant CRC patients. We plan to conduct more in-depth studies in the future.

## Conclusion

In conclusion, this study demonstrated that BRAF V600E mutation exacerbates T cell dysfunction in CRC. Furthermore, we identified core genes associated with T cell dysfunction in BRAF V600E-mutant CRC and established a novel molecular subtype. Using machine learning algorithms, we also identified IDO1 as a potential immunotherapy target for BRAF V600E-mutant CRC. These findings provide a new perspective on enhancing the efficacy of immunotherapy for this patient population.

## Data Availability

Publicly available datasets were analyzed in this study. These data can be found in TCGA (https://portal.gdc.cancer.gov/) and GEO (https://www.ncbi.nlm.nih.gov/geo/).
